# Late antiretroviral refills and condomless sex in a cohort of HIV-seropositive pregnant and postpartum Kenyan women

**DOI:** 10.1371/journal.pone.0254767

**Published:** 2021-07-19

**Authors:** McKenna C. Eastment, John Kinuthia, Lei Wang, George Wanje, Katherine Wilson, Anne Kaggiah, Jane M. Simoni, Kishorchandra Mandaliya, Danielle N. Poole, Barbra A. Richardson, Walter Jaoko, Grace John-Stewart, R. Scott McClelland

**Affiliations:** 1 Departments of Medicine, University of Washington, Seattle, Washington, United States of America; 2 Global Health, University of Washington, Seattle, Washington, United States of America; 3 Kenyatta National Hospital, Nairobi, Kenya; 4 Department of Medical Microbiology, University of Nairobi, Nairobi, Kenya; 5 Psychology, University of Washington, Seattle, Washington, United States of America; 6 Department of Geography, Dartmouth College, Hanover, New Hampshire, United States of America; 7 Biostatistics, University of Washington, Seattle, Washington, United States of America; 8 Epidemiology, University of Washington, Seattle, Washington, United States of America; 9 Pediatrics, University of Washington, Seattle, Washington, United States of America; FHI360, UNITED STATES

## Abstract

**Introduction:**

The postpartum period can be challenging for women living with HIV. Understanding how the postpartum period impacts ART adherence and condomless sex could inform the development of comprehensive sexual and reproductive health and HIV services tailored to the needs of women living with HIV during this critical interval.

**Methods:**

In a longitudinal cohort study of HIV-seropositive Kenyan women, late ART refills and self-reported condomless sex were compared between the woman’s pregnancy and the postpartum period. Analyses were conducted using generalized estimating equations and adjusted for alcohol use, depressive symptoms, intimate partner violence (IPV), and having a recent regular partner. Effect modification was explored for selected variables.

**Results and discussion:**

151 women contributed visits. Late ART refills occurred at 7% (32/439) of pregnancy visits compared to 18% (178/1016) during the postpartum period (adjusted relative risk [aRR] 2.44, 95% confidence interval [CI] 1.62–3.67). This association differed by women’s education level. Women with ≥8 years of education had late ART refills more during the postpartum period than pregnancy (aRR 3.00, 95%CI 1.95–4.62). In contrast, in women with <8 years of education, late ART refills occurred similarly during pregnancy and the postpartum period (aRR 0.88, 95%CI 0.18–4.35). Women reported condomless sex at 10% (60/600) of pregnancy visits compared to 7% (72/1081) of postpartum visits (aRR 0.76, 95%CI 0.45–1.27). This association differed by whether women had experienced recent IPV. Women without recent IPV had a significant decline in condomless sex from pregnancy to postpartum (aRR 0.53, 95%CI 0.30–0.95) while women with recent IPV had no significant change in condomless sex from pregnancy to postpartum (aRR 1.76, 95%CI 0.87–3.55).

**Conclusion:**

Improved support for ART adherence during the postpartum period and addressing IPV to limit condomless sex could improve HIV treatment and prevention outcomes for HIV-seropositive women as well as their infants and sexual partners.

## Introduction

Globally, women of childbearing age bear a disproportionate burden of human immunodeficiency virus (HIV) infection [1–3]. Many women first receive a diagnosis of HIV infection during antenatal testing [4, 5], and significant attention has focused on supporting antiretroviral therapy (ART) initiation during pregnancy to prevent mother to child transmission of HIV [6–11]. The postpartum period brings continued challenges of adhering to ART while also caring for a new baby [12–17]. Poor ART adherence places women at risk for HIV disease progression as well as transmission to their infants and sex partners [18–23]. Multiple studies have found that consistent condom use during pregnancy is low, ranging from 4% to approximately 30% [24–27]. Resumption of sexual activity in the postpartum period typically takes place a median of 6–12 months following delivery, and only about one-third of women use condoms when they resume sexual activity [28, 29]. However, a study in Kenya found that resumption of sex following delivery was much shorter with a median time of only 7 weeks [23].

Intimate partner violence (IPV) is common in Africa, with a lifetime prevalence cited as 36% [30]. In Kenya, 39% of married women report having ever experienced IPV [31]. In addition, IPV is common during pregnancy and the postpartum period [32, 33]. Intimate partner violence has been associated with negative health consequences including more risky sexual behaviors [34], depression [35], lower ART adherence, and lower rates of plasma HIV suppression [36].

Understanding how the postpartum period impacts ART adherence and condomless sex could inform the development of comprehensive sexual and reproductive health and HIV services tailored to the needs of women living with HIV during this critical interval. The objective of this analysis was to test the hypotheses that in women living with HIV, compared to pregnancy, the postpartum period is characterized by lower ART adherence and higher rates of self-reported condomless sex. Additional analyses explored potential contributing factors to explain these findings.

## Materials and methods

This analysis was nested within the *Lifecourse Study*, a prospective cohort study examining the relationship between women’s reproductive life course events and HIV transmission risk in three groups of women recruited in Nairobi and Mombasa, Kenya: females who engage in sex work (FSW), women enrolled through prevention of mother-to-child transmission (PMTCT) programs, and HIV-seropositive women in discordant couples. HIV-seropositive women were eligible to enroll if they were 18 years or older or emancipated minors. General population women were recruited through antenatal clinics, enrolled during pregnancy, and followed until 12 months postpartum. Women in the FSW cohort and discordant couples cohort were also eligible for inclusion in this analysis if they were followed while pregnant and during the postpartum period.

On enrollment, women completed a face-to-face interview in English or Kiswahili to collect demographic, sexual, behavioral, and partner characteristics. A medical history including HIV history was collected. Depressive symptoms were assessed using the standardized patient health questionnaire-9 (PHQ-9) [37, 38], and alcohol use was evaluated using the Alcohol Use Disorder Identification Test (AUDIT) [39]. Screening for intimate partner violence (IPV) and partner controlling behaviors was conducted using an adaptation of the World Health Organization (WHO) Violence Against Women questionnaire [40, 41]. This questionnaire asks about acts of physical violence, emotional violence, sexual violence, and controlling behaviors (S1 File). A complete physical examination was performed including a pelvic examination with sample collection for diagnosis of sexually transmitted infections (STIs) and prostate specific antigen (PSA) testing. This test is a biomarker for condomless sex and detects exposure to semen in the prior 24–48 hours [42]. Vaginal examinations including genital secretion sampling for PSA detection were not performed between 7 months gestation and 6 weeks postpartum. Blood samples were collected for HIV viral load (VL) and CD4 count. Sexually transmitted infections were treated at no cost. Women received ART at the study clinics according to Kenyan National Guidelines. Participants received their routine antenatal care at antenatal clinics which followed Kenyan National Guidelines for preventing-mother-to-child-transmission of HIV and clinical VL monitoring during pregnancy.

Participants returned monthly for ART refills and assessment of adherence. Pelvic examinations and genital sample collection were performed quarterly. Screening for depressive symptoms was completed every 6 months, while screening for alcohol use, IPV, and partner controlling behavior were conducted annually. Women were asked about contraceptive use during the postpartum period.

Case report forms (CRFs) were reviewed daily to ensure that all fields were appropriately completed. Data were entered into an SPSS database. At quarterly intervals, database entries were printed and compared line-by-line to hard copy CRFs to identify and correct data entry errors.

Women were reimbursed 250 Kenyan shillings (~$2.50) at each visit to compensate for travel expenses. All women provided written informed consent to take part in this study. Ethical approval was obtained from the Kenyatta National Hospital-University of Nairobi Ethics and Research Committee and the University of Washington Human Subjects Division.

### Laboratory methods

Neisseria gonorrhoeae, Chlamydia trachomatis, and Trichomonas vaginalis were diagnosed using nucleic acid amplification testing on vaginal swabs (Aptima; Hologic, San Diego, CA, USA). Prostate specific antigen was detected in genital secretions using a rapid test for the p30 antigen (ABACard, West Hills, CA, USA). HIV ribonucleic acid quantification was performed using the Hologic/Gen-Probe 2^nd^ generation assay, with a lower limit of quantification of 30 copies/mL. Some 100 mL blood samples were diluted six-fold to 600 mLs prior to testing, so viral suppression for this study was defined as ≤180 c/mL [43]. Assessment of CD4 counts was performed using FACSCount (Becton–Dickinson, Forrest Lakes, NJ, USA).

### Statistical analyses

Women enrolled between April 2013 and October 2017 were included in this analysis. The primary exposure was the postpartum period compared to pregnancy using within-woman comparisons. For women who became pregnant more than once during the study, only the first pregnancy was included in this analysis. Late ART refill and self-reported condomless sex were the primary outcomes, assessed monthly. Late ART refill was defined as a woman refilling her ART >48 hours after she was expected to have run out of medications based on the date and quantity of her last refill [43]. Condomless sex was a binary variable based on whether the number of sex acts exceeded the number of sex acts with a condom during the past week [41]. Visits where women were abstinent in the past week were included with visits where women used condoms with 100% of sex acts. Secondary outcomes included detectable plasma HIV VL (every 6 months), PSA detection in genital secretions (quarterly), and HIV transmission potential (every 6 months), defined as visits when late ART refill and self-reported condomless sex were present at the same visit. Viral loads collected within 90 days of ART initiation date were excluded [44]. Blood draws for VL measurements used in the research study were drawn every 6 months, so if women initiated ART upon presentation to antenatal care, a second VL during pregnancy may not have been available for this analysis.

Alcohol use was categorized using AUDIT as non-drinkers (score of 0), minimal (1–6), moderate (7–15), and severe problem or possibly alcohol use disorder (score ≥16) [39]. Because this distribution was highly skewed with most women reporting no alcohol use, this variable was dichotomized as any versus no use in the past year [43]. Intimate partner violence was categorized as present if any of the 14 questionnaire items indicating emotional, sexual, or physical violence were endorsed [43]. Controlling behavior by the partner was considered to be present if any of 7 items were reported [43]. Intervals for collection of these variables were based on established recall periods for the data collection instruments. Current engagement in sex work was assessed annually. The presence of regular and casual partners in the past 3 months was assessed each quarter.

Variables that were collected annually, such as alcohol use and IPV, contributed data once during pregnancy and once during the postpartum period. For variables that were collected more frequently, the response closest to the delivery date was used.

Counts and proportions were calculated for baseline sociodemographic, behavioral, laboratory, and partner characteristics. Univariate analyses were performed to compare outcomes during the postpartum period versus during pregnancy using generalized estimating equations (GEE) with Poisson log link, robust standard errors, and independent correlation structure to generate risk ratios and 95% confidence intervals (95% CI). In analyses where GEE with Poisson log link did not converge, risk differences were calculated using linear regression GEE with robust standard errors, and independent correlation structure. Potential confounding factors were considered for inclusion in multivariable models based on the scientific question and well characterized associations from the literature. In addition, because women served as their own controls for this analysis, the potential for variables to act as confounders was considered in relation to whether they could have varied between the pregnant and postpartum periods. Variables considered for adjustment included engaging in sex work [45], alcohol use [46], depressive symptoms [47], partners’ controlling behavior, IPV [41, 43], having a casual partner in the past 3 months, and having a regular partner in the past 3 months. Because of the relatively small sample size and the numerous potential confounders, a forward stepwise model building approach was used, and variables were retained if they changed the risk ratio for the primary exposure (postpartum status) by ≥10% [48]. Alcohol use, depression, IPV in the past 12 months, and a regular partner in the past 3 months were included in final adjusted models. To identify potential effect modifiers, variables were considered based on published studies, detailed knowledge of this population, and effect modifiers identified in previous analyses in the *Lifecourse Study* [41, 43]. Stratification by these variables was performed if interaction term p-values were <0.05. Variables under consideration were age, education, marital status, engaging in sex work, alcohol use, depressive symptoms, partner’s controlling behavior, and IPV. Education level was identified as an effect modifier in the late ART analysis, and IPV was identified as an effect modifier in the condomless sex analysis. Analyses were conducted using R software [49].

## Results

Seventy-five percent (114/151) of women were recruited through PMTCT clinics, while 7.9% (12/151) were from the sex worker cohort and 16.5% (25/151) were from the discordant couples cohort (Table 1). Their median age was 31 years (interquartile range [IQR] 28–34). The majority of women had ≥8 years of education (n = 136, 90.1%) and were currently married (n = 115, 76.2%). Few women reported sex work during pregnancy (n = 22, 14.7%) or the postpartum period (n = 13, 9.6%). Alcohol use was reported by 19.3% (n = 29) of women during pregnancy and 8.1% (n = 11) of women during the postpartum period. Approximately one-third of women experienced controlling behavior by a partner during each time period (pregnancy n = 47, 31.1%; postpartum n = 39, 28.9%). Intimate partner violence in the past 12 months was reported by 32.5% (n = 49) of women during pregnancy and 21.5% (n = 29) of women during the postpartum period. Most women had a regular partner during pregnancy (n = 130/144, 90.3%), but this proportion decreased during the postpartum period (n = 101/148, 68.2%). Sexually transmitted infections were rare during both the pregnant and postpartum periods. In the postpartum period, 43.7% (66/151) of women initiated modern non-barrier contraception a median of 114 days postpartum (IQR 77–164).

**Table 1:** Sociodemographic, behavioral, partner, and clinical characteristics during of 151 women during pregnancy and the postpartum period included in this analysis.

	Pregnancy Median (IQR) / Count (%)	Postpartum Period Median (IQR) / Count (%)
***Sociodemographic Characteristics***^1^		
Age (years)	31 (28–34)	
Education (≥8 years)	136 (90.1%)	
Marital status		
Currently married	115 (76.2%)	
Never married	20 (13.2%)	
Widowed/divorced	16 (10.6%)	
Number of prior pregnancies	1 (1–2)	
***Behavioral Characteristics***		
Engaging in sex work	22/150 (14.7%)^2^	13/135 (9.6%)
Alcohol use by AUDIT		
None (0)	121/150 (80.7%)	124/135 (91.9%)
Minimal (1–6)	21/150 (14%)	6/135 (4.4%)
Moderate (7–15)	7/150 (4.7%)	5/135 (3.7%)
High (16+)	1/150 (0.7%)	0/135 (0%)
Depressive symptoms by PHQ-9		
Minimal (0–4)	118/151 (78.1%)	136/147 (92.5%)
Mild (5–9)	27/151 (17.9%)	10/147 (6.8%)
Moderate/severe (10+)	6/151 (4%)	1/147 (0.7%)
***Partner Characteristics***		
Regular partner in last 3 months	130/144 (90.3%)	101/148 (68.2%)
Casual partner in last 3 months	6/151 (4%)	1/148 (0.7%)
Controlling behaviors by index partner	47 (31.1%)	39/135 (28.9%)
Intimate partner violence in past 12 months	49 (32.5%)	29/135 (21.5%)
***Clinical Characteristics***		
HIV viral load, copies/ml (Geometric mean (SD))	238.0 (12.2)	87.3 (8.9)
Absolute number of CD4+ cells/ul	468 (314–654)	640 (494–884)
Presence of *Neisseria gonorrhoeae*	1/142 (0.7%)	1/481 (0.2%)
Presence of *Chlamydia trachomatis*	1/142 (0.7%)	3/481 (0.6%)
Presence of *Trichomonas vaginalis*	2/142 (1.4%)	2/479 (0.4%)

^1^ These variables were only collected at baseline during the pregnancy period.

^2^ 1 woman (0.7%) declined to answer.

Abbreviations: IQR = interquartile range; AUDIT: alcohol use disorders identification test; HIV: human immunodeficiency virus.

### Association between pregnancy and the postpartum period and late ART refills

In univariate analyses, late ART refills occurred at 7% (32/439) of visits during pregnancy compared to 18% (178/1016) of visits in the postpartum period (relative risk [RR] 2.40, 95% confidence interval [CI] 1.68–3.44) (Table 2). This association was similar when adjusted for alcohol use, depressive symptoms, IPV in the last 12 months, and regular partner in the last 3 months (adjusted RR [aRR] 2.44, 95% CI 1.62–3.67). Late ART refills occurred at 21% (18/113) of early postpartum visits (within 42 days) and at 17% (160/1213) of visits that occurred later in the postpartum period (p = 0.4).

**Table 2:** Association between the postpartum period and late ART refill, detectable HIV viral load, self-reported condomless sex, PSA detection, and HIV transmission potential.

	Pregnancy Period n (%)	Postpartum Period n (%)	Unadjusted Relative Risk (95% CI)	p-value	Adjusted Relative Risk^1^ (95% CI)	p-value
**Late ART refill**	**32/439 (7)**	**178/1016 (18)**	**2.40 (1.68, 3.44)**	**<0.001**	**2.44 (1.62, 3.67)**	**<0.001**
**Education <8 years**^2^	8/50 (16)	22/123 (18)	1.12 (0.56, 2.23)	0.8	0.88 (0.18, 4.35)	0.9
**Education ≥8 years**	**24/389 (6)**	**156/893 (18)**	**2.83 (1.92, 4.17)**	**<0.001**	**3.00 (1.95, 4.62)**	**<0.001**
**Detectable HIV viral load**	14/118 (12)	31/254 (12)	1.03 (0.62, 1.72)	0.9	1.34 (0.76, 2.36)	0.3
** Education <8 years**	**2/13 (15)**	**2/24 (8)**	**0.54 (0.34, 0.87)**	**0.012**	--^a^	--
**Education ≥8 years**	12/105 (11)	29/230 (13)	1.10 (0.63, 1.93)	0.7	1.46 (0.79, 2.70)	0.2
**Self-reported condomless sex**	60/600 (10)	72/1081 (7)	0.67 (0.40,1.10)	0.1	0.76 (0.45, 1.27)	0.3
**IPV in the past 12 months**^3^	14/185 (8)	24/220 (11)	1.44 (0.67, 3.12)	0.4	1.76 (0.87, 3.55)	0.1
** No IPV in the past 12 months**	**46/415 (11)**	**43/804 (5)**	**0.48 (0.26, 0.90)**	**0.023**	**0.53 (0.30, 0.95)**	**0.03**
**PSA Detection**	16/144 (11)	57/475 (12)	1.08 (0.62, 1.87)	0.8	1.33 (0.79, 2.25)	0.3
**Transmission potential**^4^	5/439 (1)	9/1014 (1)	0.78 (0.27, 2.26)	0.6	0.95 (0.32, 2.82)	0.9

Abbreviations: CI = confidence interval; HIV = human immunodeficiency virus; ART = antiretroviral therapy; IPV = Intimate partner violence.

^1^ Adjusted for alcohol use, depressive symptoms, IPV in the last 12 months, and regular partner in the last 3 months.

^2^ Stratified by education, adjusted for alcohol use, depressive symptoms, IPV in the last 12 months and regular partner in the last 3 months.

^3^ Stratified by IPV in the last 12 months, adjusted for alcohol use, depressive symptoms, and regular partner in the last 3 months.

^4^ Defined as late ART refill and self-reported condomless sex.

^a^ Model did not converge.

The increase in late ART refills during the postpartum period differed between women with <8 and ≥8 years of education (p-value for interaction<0.05). Women with ≥8 years of education experienced a dramatic increase in late ART refills between pregnancy and the postpartum period, whereas women with <8 years of education had late refills similarly in both pregnancy and the postpartum period. Specifically, women with ≥8 years of education had late ART refills at only 6% (24/389) of visits during pregnancy compared to 18% (156/893) of visits during the postpartum period (RR 2.83, 95% CI 1.92–4.17). This association was similar after adjustment for alcohol use, depressive symptoms, IPV in the last 12 months, and regular partner in the last 3 months (aRR 3.00, 95% CI 1.95–4.62). In contrast, women with <8 years of education had late ART refills at 16% (8/50) of visits in pregnancy and similarly at 18% (22/123) of visits during the postpartum period (RR 1.12, 95% CI 0.56–2.23). Again, the association was similar in adjusted analyses (aRR 0.88, 95% CI 0.18–4.35).

Of the 372 HIV VLs collected ≥3 months after ART initiation, 32% (n = 118) were during pregnancy and 68% (n = 254) were during the postpartum period. Detectable HIV VLs occurred at 12% (14/118) of visits during pregnancy and 12% (31/254) during the postpartum period (RR 1.03, 95% CI 0.62–1.72). Results were similar in adjusted analyses (aRR 1.34, 95% CI 0.76–2.36). Women with ≥8 years of education had a similar prevalence of detectable HIV VL during pregnancy (11% [12/105]) and the postpartum period (13% [29/230]) (RR 1.10, 95% CI 0.63–1.93; aRR 1.46, 95% CI 0.79–2.70). In contrast, women with <8 years of education had a decline in prevalence of detectable VL from 15% (2/13) in pregnancy to 8% (2/24) during the postpartum period (RR 0.54, 95% CI 0.34–0.87). This model did not converge when potential confounders were included.

### Association between pregnancy and the postpartum period and condomless sex

Women reported condomless sex at 10% (60/600) of visits during pregnancy compared to 7% (72/1081) of visits during the postpartum period (RR 0.67, 95% CI 0.40–1.10). After adjustment for alcohol use, depressive symptoms, and regular partner in the last 3 months, this association was similar (aRR 0.76, 95% CI 0.45–1.27). The decrease in self-reported condomless sex in the postpartum period differed between women depending on whether they had experienced IPV in the past 12 months (p-value for interaction<0.05). Specifically, self-reported condomless sex declined from 11% (46/415) of visits during pregnancy to 5% (43/804) of visits in the postpartum period in women without recent IPV (RR 0.48, 95% CI 0.26–0.90). This association was similar after adjustment for alcohol use, depressive symptoms, and regular partner in the last 3 months (aRR 0.53, 95% CI 0.30–0.95). In contrast, self-reported condomless sex did not significantly change between pregnancy (8% [14/185]) and the postpartum period (11% [24/220]) for women who had experienced recent IPV (RR 1.44, 95% CI 0.67–3.12). These results were similar in adjusted analyses (aRR 1.76, 95% CI 0.87–3.55).

While data were sparse for the first 42 days postpartum, there was no self-reported condomless sex in the 113 visits in this early postpartum period. Condomless sex was reported at 7% (160/1213) of visits later in the postpartum period (43–365 days) (p<0.001 for risk difference using GEE with linear link).

Detection of PSA occurred at 11% (16/144) of visits during pregnancy compared to 12% (57/475) of visits during the postpartum period (RR 1.08, 95% CI 0.62–1.87, aRR 1.33, 95% CI 0.79–2.25). When PSA was detected, women reported condomless sex in the past week at 35% (25/72) of visits and denied condomless sex in the past week at the other 65% (47/72) of visits. This observed underreporting of condomless sex did not differ between pregnancy (63%, 10/16 visits) and the postpartum period (66%, 37/56 visits) (p = 0.3).

Episodes of late ART refills that occur concurrently with condomless sex represent instances where there may be greater risk for transmission of HIV. These events occurred at only 1% (5/439) of visits during pregnancy and 1% of visits (9/1014) in the postpartum period (RR 0.78, 95% CI 0.27–2.26). This association was similar after adjustment for alcohol use, depressive symptoms, IPV in the last 12 months, and regular partner in the last 3 months (aRR 0.95, 95% CI 0.32–2.82).

## Discussion

In this prospective cohort study of pregnant and postpartum women, the postpartum period was associated with more than twice the risk of late ART refill compared to pregnancy. Lower ART adherence during the postpartum period could impact the health of the mother, the infant, and sex partners. Overall, the prevalence of self-reported condomless sex did not differ between pregnancy and the postpartum period. However, women who had not experienced recent IPV had significantly lower self-reported condomless sex during the postpartum period compared to pregnancy, while those with recent IPV had no significant difference in self-reported condomless sex during these periods. Only one percent of visits involved late refills concurrently with condomless sex, suggesting that the combination of ART and safer sex practices may be effective in reducing the risk of sexual transmission in this population.

These results highlight that the postpartum period can be challenging for remaining adherent to ART. This parallels the findings of other studies that have observed lower ART adherence during the postpartum period [12–15]. Strategies to address this problem have included strong psychosocial support, home visits, appointment reminders [50], and a multidisciplinary team to provide seamless care from antenatal to postnatal clinics [51]. In women with higher levels of education, late ART refills were much less common during pregnancy, but increased substantially during the postpartum period. In contrast, in women with lower educational attainment, late ART refills were common during both pregnancy and the postpartum period. The literature provides conflicting data regarding the association between educational attainment and ART adherence. Studies in South Africa, Brazil, and the US have documented higher adherence in women with higher levels of education [52–56]. In contrast, one study in South Africa found that a higher level of education was associated with missing HIV clinic visits [57]. Data from the present study of Kenyan women suggest that higher levels of education may be associated with better adherence in pregnancy, but that the effect of higher education is no longer evident during the postpartum period, when the physical, emotional, and financial stresses of having a newborn may be associated with lower adherence regardless of educational level [12–15]. Further work is needed to address this question.

In previous studies, nondisclosure of HIV status [29, 58, 59], IPV [60], and alcohol use [61] have been associated with condomless sex in the postpartum period. However, prior studies have not characterized the change in prevalence of condomless sex as women transition from pregnancy to the postpartum period. In this analysis, the overall risk of self-reported condomless sex was similar during pregnancy and the postpartum period. When comparing these periods, it is important to consider that choices about condom use may have been influenced by contraceptive choices. Women may have elected not to use condoms if they were using other non-barrier forms of contraception. While there were no differences in overall risk of self-reported condomless sex between pregnancy and the postpartum period, there were distinctly different patterns depending on whether women were experiencing IPV. In the absence of IPV in the past year, the risk of self-reported condomless sex during the postpartum period declined by approximately 50% compared to pregnancy. In contrast, when IPV was reported in the postpartum period, there was no significant decrease in self-reported condomless sex. This finding is consistent with earlier studies in which IPV was associated with condomless sex in the postpartum period [58, 60]. Because these findings were adjusted for women having a regular partner in the past 3 months, it is less likely that these findings are because of a change in partners between pregnancy and the postpartum period. Research exploring the mechanisms through which IPV may influence sexual behavior have suggested that partner controlling behaviors [60] and women’s engagement in sex work [41] may be important contributors.

Similar to the overall risk for self-reported condomless sex during pregnancy and the postpartum period, PSA detection in vaginal secretions did not differ between pregnancy and the postpartum period. However, it is important to note that self-reported condomless sex was assessed in the past week while PSA detection is a biological marker for condomless sex in the past one to two days. When comparing the results of the analyses of self-reported condomless sex with the results of PSA detection, there may be differences in the prevalence of condomless sex depending on which measure was used. Future studies could consider self-collected swabs to detect PSA that coincide with intervals for self-reported condomless sex.

There were a number of strengths of this study. Follow-up of individual women from pregnancy through the postpartum period allowed for within-participant analyses. This helped eliminate some potential biases that would occur if different populations of pregnant and postpartum women were compared. In addition, as this was a longitudinal cohort, important covariates that could change over time were collected during pregnancy and during the postpartum period, and were considered in the multivariable analyses. Finally, this study used validated and well-characterized tools including an adaptation of the WHO Violence Against Women Questionnaire, AUDIT score, and the PHQ-9 to measure important potential confounders and effect modifiers.

There were also a number of limitations. First, the primary sexual behavioral outcome, condomless sex in the past week, was collected by self-report, which is subject to recall and social desirability bias. Because vaginal examinations were not performed between 7 months gestation and 6 weeks postpartum, it was not possible to use PSA detection as the primary outcome in these analyses spanning pregnancy and the postpartum period. Nonetheless, the PSA results are helpful in contextualizing and interpreting the results of self-reported condomless sex, as they highlight substantial under-reporting of condomless sex. Second, relatively few women had an HIV VL during pregnancy that was at least 90 days after ART initiation. Many women present to antenatal care after the first trimester [62–64]. Women receiving a new HIV diagnosis and initiating ART in this setting would have had a viral load drawn at ART initiation [65]. This first viral load would have been excluded from this analysis as it was pre-ART initiation. The next HIV viral load for the research study would have been collected six months later which likely would have been during the postpartum period. There would not have been a viral load during pregnancy to use in the analysis. As a result, it did not make sense to compare viral suppression during pregnancy versus the postpartum period. Because of this limitation, late ART refill was used as the primary outcome [66]. Third, alcohol use, IPV in the past 12 months, and depressive symptoms were measured at intervals of 6 months to 1 year, so there is likely to be some misclassification when these measures are used to characterize the entire pregnancy or postpartum period. Fourth, the results highlight a significant decrease in women having a regular partner from pregnancy to the postpartum period. This is likely explained by women equating a regular partner with a regular *sex* partner. Women likely indicated they did not have a regular partner in the postpartum period if they had not resumed sexual activity following delivery. However, this does highlight a limitation in this analysis as data were not available for timing of resuming sex following delivery. Fifth, women were recruited from urban and peri-urban settings and these findings may not be generalizable to more rural populations. Finally, this was a relatively small sample of pregnant and postpartum women and clinically important differences may not have been detected for some variables.

## Conclusion

In conclusion, this study adds to a growing literature suggesting that ART adherence declines during the postpartum period. Improved support for ART adherence and continuing to support condom use by addressing IPV during this challenging period in a woman’ reproductive life course could improve HIV treatment and prevention outcomes for HIV-positive women as well as their infants and sex partners.

## Supporting information

S1 File Supporting documents. Adapted WHO violence against women questionnaire.
(DOCX)
