# Supplementary material for: Late antiretroviral refills and condomless sex in a cohort of HIV-seropositive pregnant and postpartum Kenyan women
Source: PLoS One. 2021 Jul 19;16(7):e0254767. doi: 10.1371/journal.pone.0254767 (PMC8289061; doi:10.1371/journal.pone.0254767)

**S1 File**

**Adapted WHO Violence Against Women Questionnaire.**

**I want you to think about your current or most recent regular male sex partner. This could be a husband or a partner with whom you have (or had) a committed relationship.** *Prompt:* **This is not someone with whom you have only had causal sex or sex in exchange for money or gifts. Some women may not have had a regular partner.**

1. Do you have a current or most recent regular partner?
2. During the last three months, have you had a regular partner?

**Next, I would like to ask some questions about your current and past relationships, and how your husband/male partner treats (treated) you. Please answer these questions about the same person you identified as a current or most recent regular male sex partner earlier.**

**Thinking about your current or most recent regular partner, would you say it is generally true that he:**

1. Tries to keep you from seeing your friends?
2. Tries to restrict contact with your family of birth?
3. Insists on knowing where you are at all times?
4. Ignores you and treats you indifferently?
5. Gets angry if you speak with another man?
6. Is often suspicious that you are unfaithful?
7. Expects you to ask his permission before seeking health care for yourself?

**The next questions are about things that happen to many women and that your current or most recent regular partner may have done to you. Has your current regular or most recent regular partner:**

1. Insulted you or made you feel bad about yourself?
2. Belittled or humiliated you in front of other people?
3. Done things to scare or intimate you on purpose, e.g. by the way he looked at you, by yelling or smashing things?
4. Threatened to hurt you or someone you care about?
5. Slapped you or thrown something at you that could hurt you?
6. Pushed or shoved you or pulled your hair?
7. Hit you with his fist or with something else that could hurt?
8. Kicked you, dragged you, or beaten you up?
9. Choked or burnt you on purpose?
10. Threatened to use or actually used a gun, knife, or other weapon against you?
11. Physically forced you to have sexual intercourse when you did not want to?
12. Did you have sexual intercourse you did not want to because you were afraid of what he might do?
13. Did he force you to do something sexual that you found degrading or humiliating?
14. Have you been injured as a result of these acts by your current regular partner?

Please think of the acts that we talked about before.

**Since the age of 15 years has anyone (other than your current or most recent regular partner):**

*Responses for questions 24 and 25:*

*0=never; 1=once or twice; 2=a few times; 3=many times*

1. Beaten or physically mistreated you in any way?
2. Forced you to have sex or perform a sexual act when you did not want to?
3. Who did this to you? ***Enter 0=no or 1=yes for each response:***


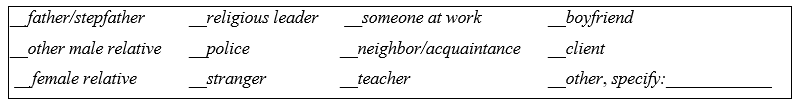

Supplement: S1 File — Adapted WHO violence against women questionnaire. (DOCX) [file pone.0254767.s001.docx]
